# Refugee Telehealth Utilization for Hypertension Management During the COVID-19 Pandemic

**DOI:** 10.3389/ijph.2023.1605913

**Published:** 2023-08-08

**Authors:** Zaid Salameh, Dahlia A. Kaki, Dania Abu Baker, Tarek Hijazi, Job Godino, Tala Al-Rousan

**Affiliations:** ^1^ Mailman School of Public Health, Columbia University, New York City, NY, United States; ^2^ School of Medicine, University of California, San Francisco, San Francisco, CA, United States; ^3^ Herbert Wertheim School of Public Health and Human Longevity Science, University of California, San Diego, La Jolla, CA, United States; ^4^ Family Health Centers of San Diego, San Diego, CA, United States

**Keywords:** refugee health, telehealth, COVID-19, hypertension management, health equity

## Abstract

**Objectives:** The COVID-19 pandemic limited refugees’ access to healthcare. Increased use of telehealth could enable continuity of care but also create barriers to chronic disease management. This study explores refugees’ experience with telehealth and hypertension management during the pandemic.

**Methods:** We recruited 109 refugee participants diagnosed with hypertension. We conducted semi-structured interviews about their experience with telehealth during the COVID-19 pandemic. Interviews were transcribed, translated, and data was coded using inductive thematic analysis.

**Results:** 86% used telehealth modalities at least once during the pandemic. Interviews highlighted three main themes: (1) Social isolation worsened mental health, affecting their motivation to manage their blood pressure; (2) telehealth alleviated discontinuity of care but posed logistical and cultural challenges; (3) participants relied on public blood pressure monitors that were not available during the pandemic which affected disease management.

**Conclusion:** Refugees faced challenges managing their hypertension during the COVID-19 pandemic. Virtual community building may alleviate their stress and isolation. Telehealth must be adapted to account for language, cultural, and technological barriers. Communities with hypertension should increase access to personal or public blood pressure monitors.

## Introduction

While the impact of the COVID-19 pandemic was global, the burdens of the virus were not shared equally. These discrepancies were most evident in the high death and case rate amongst racial and ethnic minorities in the United States [1]. Refugees, in particular, experienced high rates of morbidity and mortality compared to native-born Americans in part because of their tenuous access to healthcare services [2–4]. Refugees in the United States are also more likely to work in frontline roles, including transportation, retail, and hospitality, increasing their risk of exposure and disease [2, 4, 5].

Refugees have been shown to be more susceptible to persistent health disparities in cardiovascular disease outcomes [6–8]. Cardiovascular disease continues to be the most prevalent disease among refugees, with uncontrolled blood pressure being the most prominent risk factor [9, 10]. In addition, foreign-born individuals are more likely to be unaware of their hypertension [11]. In the context of a pandemic, this places refugee patients with hypertension at higher risk for COVID-19 complications. The pandemic has exacerbated blood pressure control among many adults in the United States, with disparities in hypertension management amongst ethnic and racial minorities [12, 13]. However, there is little to no literature specifically about refugees’ blood pressure control during the pandemic.

The pandemic’s most notorious benefit, however, was the advent of telehealth modalities, particularly in outpatient settings [14]. Telehealth has a long history of utility in emergency settings, including during the 2003 SARS outbreak, Australia’s 2020 fire season and drought, and in the United States during Hurricane Irma and Harvey [15–18]. Telehealth facilitates access to primary and mental healthcare for rural communities and ensures continuity of care in times of crisis. Telehealth has also proven to be a healthcare access equalizer. For many patients, telehealth is cheaper, requires less time off work, and limits transportation inconveniences [19]. For patients experiencing chronic illnesses that require frequent provider visits, like diabetes or hypertension, telehealth can reduce patient burden and time-spent receiving care. During a pandemic, minimizing contact with healthcare systems proved to be critical for both providers and high-risk patients [20, 21].

Nevertheless, like all healthcare innovations, telehealth modalities do have their limitations. Prior research demonstrates that access to technology is not equal and race, geography, socioeconomic status, and age disparities play a significant role in the efficacy of widespread telehealth use [22–25]. While little literature exists highlighting the telehealth experience of refugee populations specifically, refugees often fit into the same sociodemographic groups that have been documented to experience telehealth disparities. The majority of refugee patients relying on telehealth are older, less technologically literate, and more likely to be limited-English proficiency patients, limiting their ability to engage with telehealth care [26].

As global mass displacement increases and refugee resettlement grows, it is important to understand this population’s access to and engagement with healthcare systems, particularly as it applies to chronic disease management. San Diego, California is the second-largest resettlement city in the United States, with immigrants, including refugees, making up 21.5% of the population [5]. This study aims to document United States-resettled refugees’ experience with telehealth modalities for hypertension management during the peak of the COVID-19 pandemic and to close the literature gap on refugee health experiences with this growing healthcare technology. Specifically, this qualitative study assesses the impact of the COVID-19 pandemic on patients’ hypertension management and their uptake of telehealth tools during the pandemic.

## Methods

This study is part of a larger effort to better understand refugee patients’ barriers to hypertension care and improve access to self-management tools. Participants were provided a free *Withings BPM Connect*, an FDA-approved cellular-connected at-home blood pressure monitor, and were instructed to maintain a blood pressure diary. With the onset of the COVID-19 pandemic, the study sought to understand the participants’ experience with the provided technology and their hypertension management generally, as many switched to telehealth models of care. Ethical approval to conduct the study was obtained through the University of California, San Diego.

### Setting

The American healthcare system is made up of healthcare providers, insurance companies, government programs, and regulatory bodies [27]. Through premiums, deductibles, copays, and co-insurance, healthcare expenses are frequently split between individuals, employers, and insurance companies [27]. The majority of Americans have access to private health insurance through privately purchased or employer-provided plans [28]. Government-funded health insurance plans, like Medicare and Medicaid, are also available for the elderly and people with low-income status [27, 28]. The US Refugee Resettlement Program provides initial primary care services including vaccinations, initial medical screenings, and referrals to appropriate healthcare providers [29, 30]. Refugees may be eligible for medical aid through the Refugee Medical Assistance (RMA) program during their first 8 months in the US or until they are eligible for government-funded health insurance plans [29]. Healthcare access can also greatly vary based on location, available resources, and individual circumstances [31].

There are significant difficulties and disparities associated with healthcare access, cost, service quality, and disparities for patients using government-funded healthcare services [31]. The COVID-19 pandemic has further exacerbated these disparities, specifically within refugee and immigrant populations [32]. Refugees globally are more likely to experience poverty compared to the native-born population which is an important social determinant of healthcare access [33–35]. In the US specifically, refugees are twice as likely to be unemployed [36]. Other factors like literacy and education level, pre-migration socioeconomic status, and social support access affect the refugee resettlement experience in the US and create additional barriers to healthcare access [37–40].

### Participants

Participants were Syrian and Iraqi refugee patients at the Family Health Center of San Diego (FHCSD) with a diagnosis of hypertension. We recruited the participants by e-mailing study pamphlets through an ethnic-based community organization Majdal Community Center in El Cajon, San Diego) and contacting patients through the FHCSD patients’ list. Members of the research team contacted patients by phone or email after their visits to FHCSD asking if they may be interested in participating. Participants were consented and scheduled for either an in-person interview at the Majdal Community Center or a virtual interview via Zoom, Skype, or phone call.

### Data Collection

Semi-structured interviews, ranging from 30 to 90 min, were conducted before the distribution of the blood pressure monitors by bilingual Arabic-speaking investigators between April 2021 and April 2022. The interview guide (Table 1) was developed and vetted by field experts and direct providers. Interviews explored attitudes towards telehealth modalities for hypertension management among refugee patients including barriers and facilitators to home blood pressure management. Interviews were conducted in Arabic on a password-protected video-conferencing platform or in-person at the Majdal Community Center. Interviews were audio-recorded, transcribed in Arabic, translated into English, and reviewed for content and accuracy.

**TABLE 1:** Interview Guide used to examine barriers and facilitators to accessing telehealth during the COVID-19 among hypertensive refugees (San Diego, USA. 2020).

1. Due to the COVID-19 pandemic, did you decide not to:
a. Attend a scheduled in-person general medical appointment not cancelled due to the COVID-19 pandemic?
b. Seek emergency care in an urgent care facility or emergency room?
2. Did you participate in a telehealth medical appointment (e.g., Zoom, Facetime) since the COVID-19 pandemic started?
3. If you had a telehealth appointment, how satisfied are you with your experience?
4. Does high blood pressure worry you? Tell me why
5. Do you get your blood pressure regularly checked?
6. What are the difficulties in getting your blood pressure checked regularly?
7. Are you taking medications for your blood pressure? What are the things that make it difficult for you to take your medicine? What are the things that could make it easier for you to take your medications?
8. How would you describe your relationship with your doctor and your experiences with the healthcare system in this country? What can improve this experience?
9. In what ways has the refugee experiences impacted your hypertension management and your overall health including social relationships?
10. What would make life better for people with high blood pressure? Any final thoughts for what you think may achieve good blood pressure control?

### Data Analysis

We used an inductive thematic analysis to analyze the transcripts. Three members of the research team independently reviewed and coded each interview transcript using QSR NVivo V10 software. Analysis was done through the identification of recurrent themes following Crabtree and Miller’s 5-step interpretive process [41]. Members of the research team worked collaboratively to build a codebook before analyzing the data for broad categories or themes.

## Results

We had 109 participants, with 77.9% Iraqis (N = 85) and 22.0% Syrians (N = 24). 56 were male-identifying and 53 were female-identifying. The average participant age was 61 years old and the average length of residence in the United States was 9.6 years. Additional demographic information, including educational, employment, and relationship status are listed in Table 2. 45% of participants did not attend medical appointments and 22% did not seek urgent care despite needing it due to COVID-19 restrictions. 86% used telehealth modalities at least once since the start of the pandemic.

**TABLE 2:** Participant demographics and healthcare use during the COVID-19 pandemic (San Diego, USA. 2020).

	Iraqi *n =* 85	Syrian *n =* 24	All *n = 109*
Age, years (mean, SD)	63 (9.2)	53 (7.6)	61 (9.7)
Years in the U.S (mean, SD)	10.6 (6.2)	6.0 (1.3)	9.6 (5.8)
Gender			
Female	44 (51.7)	9 (37.5)	53 (48.6)
Male	41 (48.3)	15 (62.5)	56 (51.4)
Marital Status			
Married	70 (82.4)	22 (91.7)	92 (84.4)
Divorced	3 (3.5)	0	3 (2.75)
Widowed	11 (12.9)	2 (8.3)	13 (11.9)
Never Married	1 (1.2)	0	1 (.95)
Highest level of education			
Less than high school	29 (34.1)	13 (54.1)	42 (38.5)
High school	18 (21.2)	9 (37.5)	27 (24.7)
Vocational certificate	15 (17.6)	1 (4.2)	16 (14.7)
Undergraduate	18 (21.2)	0	19 (17.5)
Postgraduate	5 (5.9)	1 (4.2)	5 (4.6)
Employed			
Yes	13 (15.3)	2 (8.3)	15 (13.8)
No	72 (84.7)	21 (87.5)	93 (85.3)
No Response	0	1 (4.2)	1 (.9)
Annual income			
Less than $15,000	49 (57.6)	18 (75.0)	67 (61.5)
$15,001–$25,000	26 (30.6)	5 (20.8)	31 (28.5)
$25,001–$35,000	7 (8.2)	0	7 (6.4)
$35,001–$50,000	2 (2.4)	0	2 (1.8)
$50,000+	1 (1.2)	1 (4.2)	2 (1.8)
Proficient in English			
Yes	33 (38.8)	5 (20.8)	38 (34.9)
No	52 (61.2)	19 (79.2)	71 (65.1)
Ever tested positive for COVID-19			
Yes	23 (27.1)	4 (16.7)	27 (24.8)
No	61 (71.7)	19 (79.1)	80 (73.4)
Don’t Know	1 (1.2)	1 (4.2)	2 (1.8)
Did not seek emergency medical attention due to COVID-19 pandemic			
Yes	16 (18.8)	8 (33.3)	24 (22)
No	69 (81.2)	16 (66.7)	85 (78)
Did not attend scheduled medical appointment due to COVID-19 pandemic			
Yes	39 (45.9)	10 (41.7)	49 (45)
No	46 (54.1)	14 (58.3)	60 (55)
Utilized telehealth modalities during COVID-19 pandemic			
Yes	74 (87.0)	20 (83.3)	94 (86.2)
No	11 (13.0)	4 (16.7)	15 (13.8)
Number of telehealth visits in the last 12 months	462	142	604

In the interviews, participants overwhelmingly expressed being affected by the COVID-19 pandemic. Their discussion of the pandemic and its consequences on hypertension management yielded three main themes: (1) social isolation worsening psychosocial stress; (2) limited access to healthcare providers; and (3) limited access to necessary tools for hypertension management, specifically blood pressure measuring devices.

### Theme 1: Isolation

All participants shared the heavy toll the pandemic took on their mental health. Many expressed how they relied on non-familial communities and social activities as a source of sustenance, a resource they were cut off from during the COVID-19 pandemic. For instance, one participant emphasized:

“With the pandemic halting everything and all activities, I realized how important activities are.” (Participant 49)

Spending time outside of the home, in community centers for example, and engaging in activities is a way to connect with others, cultivate new friendships, and gain social capital. For many refugees, this practice is essential to acclimating to a new environment and developing permanent roots in places where they often do not have extended family [42, 43]. Given that socializing depended on the use of public spaces, the closing of many community centers perpetuated feelings of isolation. Unable to host people in their own homes and unable to safely congregate in public spaces, the participants experienced a loss of belonging compounded by the loss of access to those who were most likely to understand their unique struggles and circumstances. An Iraqi participant highlighted this reality by explaining:

“[The pandemic] decreased our social life, since we do not know many people here and most of our families and relatives are in Iraq. Also, the pandemic affected our social life and meeting new people.” (Participant 28)

“We go to the center here and see many Iraqis, however since the pandemic they closed it and it has been a whole year without seeing any of our Iraqi friends in the U.S.” (Participant 23)

As a result of this isolation, many participants noticed they experienced worse mental health, including anxiety and acute stress. Some noted that as a result, they also experienced higher blood pressure readings and worse hypertension-associated symptoms like headaches. For example, one participant stated:

“I noticed that my hypertension was exacerbated when the pandemic started, and I was stuck at home with anxiety.” (Participant 47)

Another participant also mentioned:

“The pandemic caused me so much anxiety and forced me to not leave my house and sometimes cancel my doctor's appointments. All of this mental distress is bad for hypertension.” (Participant 23)

For some, however, the fear of being in a “high risk” category due to their hypertension diagnosis motivated them to be more engaged in their disease management. Many shared that they became more vigilant about monitoring their blood pressure and being more compliant with their hypertension medication:

“After the pandemic started, I became more paranoid about my health, so I ended up taking my medication regularly.” (Participant 10)

For these participants, the stress and anxiety of an unpredictable pandemic situation prompted them to focus on things that were within their control, including the lifestyle and medication changes needed to manage their high blood pressure.

### Theme 2: Limited Access to Healthcare Providers

As with many underserved patient groups, the pandemic limited access to healthcare providers for refugees. For the participants, this translated into worse management of their chronic disease and resulting hypertension symptoms. Many participants described not being able to access their providers. And even when they had established primary care providers, most discussed long-wait times and burdensome scheduling practices as a result of the pandemic backlog:

“When I moved to El Cajon, the pandemic started so I still do not have a doctor that I see regularly.” (Participant 32)

“With the pandemic, appointments are getting more difficult to be scheduled and they are too far away.” (Participant 51)

One participant described how even during a situation of uncontrolled hypertension; they were not able to seek immediate specialist care:

“During the pandemic, I went to the clinic to measure my blood pressure and it was high. So, the doctor referred me to a cardiologist who refused to see me on the phone probably due to the limited availability.” (Participant 3)

On the other hand, the pandemic did lead to the normalization of telehealth modalities. The participants reported engaging with telehealth tools for most of their care during the pandemic:

“With the pandemic, most of my appointments are telehealth on the phone.” (Participant 10)

“Since the pandemic started my appointments became more telehealth.”(Participant 32)

The use of telehealth in this population helped mitigate many of the access issues created by the COVID-19 pandemic by providing an alternative to in-person consultations. For those with hypertension, this was an important option to minimize their exposure to the COVID-19 virus in healthcare settings.

Nevertheless, as with all healthcare innovations, telehealth presents its own set of limitations. For many refugees, technological literacy is limited and access to electronic devices and reliable internet can be tenuous. Further, many of the participants expressed that telehealth is a poor substitute for in-person care and prefer face-to-face interactions with their providers:

“Since [the pandemic], it is all telehealth, and I can’t see the doctor face to face. Even though our doctor is super helpful.” (Participant 49)

### Theme 3: Access to Blood Pressure Measuring Devices

The participants expressed a strong reliance on public health devices (e.g., pharmacy-based blood pressure monitors). Pre-pandemic, those devices were both publicly accessible and free of charge making them convenient options for low-income refugees who needed to monitor their blood pressure. However, with public health concerns, many were no longer accessible:

“I used to go to Walgreens and use their public device, it was free. […]. [They] stopped [access to blood pressure monitors] due to the pandemic.” (Participant 35)

For some participants, this limitation prompted them to purchase their own home blood pressure monitors. Many who bought personal devices (or had access to one through this study) felt encouraged to monitor their blood pressure more regularly and take better control of their hypertension management:

“Yes, I do. Before the pandemic I used to use the pharmacy’s device to measure my blood pressure, but now I bought a device from Walmart, and it is great.” (Participant 3)

## Discussion

This study explores refugees’ perspectives on the effect of the COVID-19 pandemic and telehealth modalities on their hypertension management. As this was a qualitative study and not meant to be generalizable, it is acknowledged that this is a limitation. Thus, this limitation is specifically addressed and supported by academic literature in the paper [44]. The findings are summarized by three main themes: (1) social isolation worsened mental health, affecting participant’s blood pressure and their motivation to manage it; (2) telehealth alleviated discontinuity of care but posed significant logistical and cultural challenges; (3) participants relied on public blood pressure monitors that were not available during the pandemic which affected disease management.

For many of the participants, the pandemic’s main consequence was disrupting their access to social networks, a critical part of their daily routines. Isolation worsened anxiety and stress in a population already at risk for mental illness [45, 46]. Studies in older adults piloted the use of tele-socializing modalities and were shown to increase the quality of life outcomes and resiliency during the COVID-19 pandemic [47, 48]. The participants overwhelmingly reported the use of telehealth modalities, indicating a relative comfort with online platforms. This model can thus be applied to the refugee population who often depend on the community not only for mental wellbeing but also for integration and adaptation to the host country. Programs through community centers, such as movie showings or game nights, can help maintain this critical sense of connection. Programming to teach and encourage psychological and spiritual wellbeing may also assist in curtailing the spread of anxiety and depression during challenging times [48–51]. Primary care providers and local advocacy groups have been shown to be effective educators and may be best suited to adapting teachings to the local refugee group’s culture and language [52].

The findings also confirm prior literature that describes the double-edged nature of telehealth modalities as both an equalizer in improving access to care and as a barrier for minority patients with limited resources [22–25, 53]. We reinforce the notion that telehealth increases access to healthcare by requiring less transportation time and leave from work to attend medical appointments. However, uptake of telehealth depends on technological literacy, access to a reliable internet connection, and access to electronic devices which may not be the case for all low-income or older patients [26, 53, 54]. As healthcare systems work towards mainstreaming telehealth services outside of the pandemic context, it is important to ensure that we preserve equal access to care. For example, insurance systems or government budgets can subsidize the cost of internet subscriptions or reimburse purchases of electronic devices needed for healthcare use [55, 56]. The findings also highlighted the variation in cultural acceptance of telehealth, with many ethnic and racial minorities deeming telehealth as an unreliable or less legitimate healthcare service [57]. Healthcare providers using telehealth modalities should be trained on how to guide refugee patients through online platforms and healthcare systems can provide patient-facing training to ensure the quality of care [58]. We also encourage ongoing language and cultural competency training as they help build the patient-provider trust needed to encourage telehealth uptake in communities with telehealth skepticism. This should be done in addition to highly collaborative and interdisciplinary team-based care that has been shown to enhance the quality of services given to refugee patients.

Finally, the results emphasized the importance of patient access to public blood pressure monitors for effective hypertension management. For patients unable to afford personal devices or unable to receive one through their healthcare plan, access to pharmacy devices allowed them to maintain a sense of agency and control over their health. Pharmacies should consider offering free blood pressure screening services and in communities with a high burden of hypertension, local community centers, public offices, and religious centers can consider investing in accessible blood pressure monitors. The participants were also enrolled in a larger study, giving them access to personal blood pressure monitors, which could enable disease management. Health insurance companies, state agencies, and federal agencies may consider providing low-income patients with free personal blood pressure monitors as part of hypertension management programs.

While the research has many strengths, it is important to recognize that the participant group was limited to refugees in San Diego, California, a region that may not be representative of experiences and perspectives in other US and international resettlement cities with different policies, access levels, or ethnic and racial makeups. The first limitation as mentioned at the beginning of the discussion is that qualitative research rarely seeks to be representative [44]; rather, the study seeks to capture and elevate the narratives and perspectives of the participant population. This study serves as exploratory work upon which future studies can be built so policies and interventions can be informed. A second limitation is the study population. While the participants’ country of origin, habits, and cultures imply very natural behaviors and choices that are specific to them, the differentiation of the participant groups could have been explained more thoroughly. Overall, further investigation ought to be conducted to assess rates of telehealth uptake in global refugee populations and outcomes of blood pressure control using telehealth modalities.

### New Contributions to the Literature

This study is among the first to identify refugee perspectives on telehealth modalities during the COVID-19 pandemic and their impact on hypertension management. We suggest individual, community, and policy interventions that can be implemented to improve the experience of this underserved and vulnerable healthcare population.
